# Cancer Stem Cells in Thyroid Tumors: From the Origin to Metastasis

**DOI:** 10.3389/fendo.2020.00566

**Published:** 2020-08-25

**Authors:** Veronica Veschi, Francesco Verona, Melania Lo Iacono, Caterina D'Accardo, Gaetana Porcelli, Alice Turdo, Miriam Gaggianesi, Stefano Forte, Dario Giuffrida, Lorenzo Memeo, Matilde Todaro

**Affiliations:** ^1^Department of Surgical, Oncological and Stomatological Sciences (DICHIRONS), University of Palermo, Palermo, Italy; ^2^Department of Health Promotion, Mother and Child Care, Internal Medicine and Medical Specialties (PROMISE), University of Palermo, Palermo, Italy; ^3^Department of Experimental Oncology, Mediterranean Institute of Oncology (IOM), Catania, Italy

**Keywords:** cancer stem cells, thyroid tumors, epigenetic alterations, microenvironment, immune system

## Abstract

Thyroid tumors are extremely heterogeneous varying from almost benign tumors with good prognosis as papillary or follicular tumors, to the undifferentiated ones with severe prognosis. Recently, several models of thyroid carcinogenesis have been described, mostly hypothesizing a major role of the thyroid cancer stem cell (TCSC) population in both cancer initiation and metastasis formation. However, the cellular origin of TCSC is still incompletely understood. Here, we review the principal epigenetic mechanisms relevant to TCSC origin and maintenance in both well-differentiated and anaplastic thyroid tumors. Specifically, we describe the alterations in DNA methylation, histone modifiers, and microRNAs (miRNAs) involved in TCSC survival, focusing on the potential of targeting aberrant epigenetic modifications for developing novel therapeutic approaches. Moreover, we discuss the bidirectional relationship between TCSCs and immune cells. The cells of innate and adaptive response can promote the TCSC-driven tumorigenesis, and conversely, TCSCs may favor the expansion of immune cells with protumorigenic functions. Finally, we evaluate the role of the tumor microenvironment and the complex cross-talk of chemokines, hormones, and cytokines in regulating thyroid tumor initiation, progression, and therapy refractoriness. The re-education of the stromal cells can be an effective strategy to fight thyroid cancer. Dissecting the genetic and epigenetic landscape of TCSCs and their interactions with tumor microenvironment cells is urgently needed to select more appropriate treatment and improve the outcome of patients affected by advanced differentiated and undifferentiated thyroid cancers.

## Thyroid Tumors and Cancer Stem Cells

Thyroid cancers (TCs) are highly heterogeneous and represent the most frequent tumors among the endocrine neoplasms (1, 2). In the past years, TC incidence increased worldwide, and the gender disparity became more pronounced, especially considering women at age 40–49 with a female/male ratio of about three times higher (3). The molecular basis of these gender differences in TC is still unclear. However, it has been postulated that female steroid hormones play a critical role in TC development mediated by the differential expression of the nuclear α- and β-estrogen receptors in various TC histological subtypes (4). Moreover, estrogens increase adherence, invasion, and migration capability of thyroid cancer cell lines (5). Recently, it has been demonstrated that estradiol regulates a higher production of reactive oxygen species (ROS) in adult femal rats compared with male counterparts (6, 7). Overall, the risk of thyroid proliferative diseases is increased during pregnancy, while the specific risk for TC is decreased after menopause.

**Figure 1:**
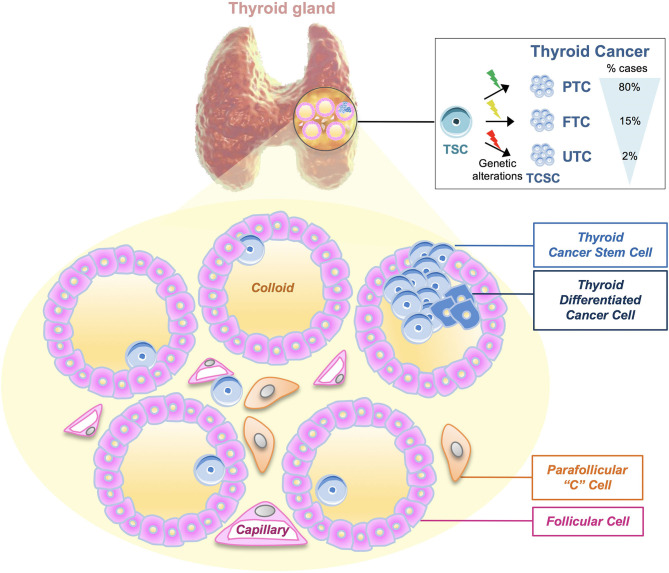
Thyroid tumors and thyroid cancer stem cell genetic model. Thyroid cancers (TCs) are heterogeneous diseases varying from well-differentiated [papillary thyroid carcinomas (PTCs), follicular thyroid carcinomas (FTCs)] to undifferentiated tumors (UTCs). According to the most recent thyroid cancer stem cell model, the cell of origin of TC may be represented by the same cancer stem cell, which is subjected to different types of genetic mutations, leading to the development of PTC (80% of cases), FTC (15%), and UTC (2%), respectively. The model shown represents the microscopic structure of thyroid follicles, in which walls of follicular cells surround the follicle lumen filled with colloid, the center of thyroid hormone production. Moreover, following this model, thyroid cancer stem cells (TCSCs) along with thyroid differentiated cancer cells may populate the thyroid follicle. Parafollicular “C” cells are the cells of origin of medullary thyroid carcinoma (MTC).

According to their histopathological features, it is possible to distinguish four subtypes of thyroid carcinoma: papillary thyroid carcinoma (PTC), follicular thyroid carcinoma (FTC), anaplastic thyroid carcinoma (ATC), and medullary thyroid carcinoma (MTC). PTC, FTC, and ATC derive from malignant transformation of follicular cells, while MTC derives from calcitonin-producing parafollicular C cells. PTCs and FTCs represent the majority of differentiated TCs with good prognosis, accounting for 80–85 and 10–15% of all TCs, respectively. On the contrary, ATC is a rare and undifferentiated TC (UTC), characterized by an aggressive phenotype and poor prognosis. Although current therapeutic strategies including surgery, radioiodine therapy, and chemotherapy are able to eradicate the majority of primary TCs, the management of advanced and undifferentiated TCs is still a clinical hurdle. The existence of cancer stem cell (CSC) population explains the aggressiveness of TCs and their resistance to the clinical treatments. Scientific advances in stem cell biology have paved the way to a better understanding of the molecular mechanisms driving tumorigenesis in many types of cancers, including TCs (8) (Figure 1). CSCs are a small subset of cancer cells within tumors that exhibit exclusive self-renewal ability, clonogenic, and metastatic potential. They show a key role during the initiation, progression, drug resistance, and cancer recurrence or metastasis (9, 10). The isolation and characterization of thyroid cancer stem cell (TCSC) population in different thyroid tumors improved the knowledge about TC initiation. Nevertheless, there are still many questions to elucidate: (i) how TCSCs influence the initiation, progression, and metastasis within the four subtypes of TCs; (ii) how the tumor microenvironment (TME) affects TCSCs; (iii) which are the interactions between TCSCs and tumor bulk population; and (iv) which is the broad genetic/epigenetic landscape of TCs. Here, we provide an overview of the principal markers and pathways sustaining TCSC survival, their epigenetic alterations and interactions with immune cells, and TME cell components. We summarize the potential and innovative therapeutic approaches targeting TCSCs. Nowadays, dissecting the role of TCSCs in TC initiation, progression, and invasiveness may lead to the development of more effective therapies in advanced TCs.

### Thyroid Cancer Model of Origin

Different carcinogenesis models have been proposed to describe TC origin. According to the multistep carcinogenesis model, ATC cells derive from FTC or PTC cells following a dedifferentiation process and the accumulation of different mutations, particularly the inactivating mutations of *TP53* and *CTNNB1* (11, 12). This model is supported by scientific evidence, such as the presence of *TP53* and *BRAF* mutations in differentiated and undifferentiated carcinomas, including ATCs (13), but it cannot explain the presence of specific *RET/PTC* rearrangements and *PAX8/PPAR*γ gene fusion that may occur in ATCs (14). Moreover, the slow cell cycle of follicular thyroid cells reduces the potential accumulation of mutations, which sustain cancer progression (15). Thus, according to the multistep carcinogenesis model, TCSCs can derive from thyrocytes upon genetic alterations and the epithelial–mesenchymal transition (EMT) process, which induces in TCSCs a more aggressive phenotype able to give rise to metastasis (16). During EMT, TCSCs lose polarity and adhesion, thus acquiring an invasive phenotype through Snail upregulation, which impairs E-cadherin expression (17). The fetal cell carcinogenesis model foresees that thyroid cancer cells derive from thyroid fetal cells upon the acquisition of specific mutations. The term thyroid fetal cells refers to TCSCs or thyroid precursor cells such as thyroblasts or prothyrocytes (18). TCs present a different cell of origin as well as a different mutational profile based on their distinct histopathological features. Specifically, ATC originates from fetal TCSCs expressing onco-fetal fibronectin without the expression of any differentiation markers, while PTC originates from thyroblasts expressing onco-fetal fibronectin and differentiation markers as thyroglobulin (Tg). Finally, FTC originates from the prothyrocytes, representing a more differentiated thyroid cell (19). Interestingly, to date, many evidence support the CSC model also for TC origin and initiation (20). CSCs represent a small population in the tumor bulk. They are placed at the apex of the hierarchical pyramid that includes also progenitors and differentiated cells. CSCs are able to drive cancer initiation and progression by acquiring genetic mutations and epigenetic alterations (21). Nowadays, the dynamic CSC model superseded the CSC model. Indeed, CSC phenotype has been considered plastic and dynamic, able to interchange between a CSC state and a non-CSC state, spontaneously or in response to microenvironment stimuli (EMT or IL-6) in various cancer types (22–24). An extensive description of the thyroid carcinogenesis models can be found in (25). Given that all the above-described models do not completely explain the phenotypic and genetic heterogeneity of tumor bulk, it has been recently postulated the genetic mutation model to elucidate the molecular mechanisms underlying the distinct TC histopathology and behavior. Specifically, according to this model, a variety of genetic mutations may occur in the same CSC by leading to different tumor phenotypes. This can be termed as the CSC genetic mutation model in TC (Figure 1).

### Normal vs. Thyroid Cancer Stem Cells

To date the origin of TCSC population is still incompletely understood. Specifically, whether TCSCs originate from precursors or mature cells and whether TC cells are the result of genetic mutations or epigenetic alterations occurring in thyroid stem cells (TSCs) are issues to be clarified. Nevertheless, the existence of TCSCs is confirmed by *in vitro* thyrosphere generation and by the development of *in vivo* mouse models. To date, several markers have been proposed to identify TCSCs, such as CD133, CD44, and aldehyde dehydrogenase gene (ALDH) (20, 26). Several studies highlighted that TCSCs present specific features that distinguish them from normal TSCs. Both CSCs and stem cells (SCs) undergo symmetric division, but their clonogenic and differentiative potential is different. Giani et al. observed that thyrospheres generated from PTC-derived CSCs (PTC-CSCs) were larger and irregular compared with normal TSCs. The clonogenic potential and expression levels of stemness markers (Oct-4, Sox-2, ABCG2) and EMT markers, as vimentin, were higher in PTC-CSCs compared with normal TSCs. Moreover, TCSCs showed lower expression levels of differentiation markers such as Pax-8 and TTF-1, and their differentiation efficiency was poorer than normal TSCs (27). Malguarnera et al. dissected the differences between CSCs derived from PTC and thyroid normal stem/progenitor cells. Both cellular types are able to generate thyrospheres in culture. However, only thyrospheres derived from normal thyroid stem progenitor cells could differentiate when plated in adhesion in presence of thyroid-stimulating hormone (TSH). Stemness markers as CD133, CD44, Oct-4, Sox-2, and Nanog were revealed in both normal and cancer thyrospheres; conversely, thyroid differentiation markers [thyroperoxidase (TPO), thyroglobulin (Tg), thyroid-stimulating hormone receptor (TSH-R)] were detected at low levels in both cellular types. In addition, the authors showed that insulin receptors (IR-A and IR-B), insulin growth factors (IGF-I and IGF II), and the IGF receptor (IGF–IR) were expressed at higher levels in CSCs compared to the differentiated cells. These findings confirm that insulin resistance is related to an enhanced susceptibility to develop TC, and therefore, insulin and/or IGFs should be considered as novel potential targets for TC treatment (28).

### Markers Identifying TCSCs and Pathways Sustaining Their Maintenance

Many studies have been carried out to identify specific biomarkers of TCSCs in the four histopathological TC variants, but their combination and/or association needs to be further investigated. Friedman et al. demonstrated that cell lines derived from ATCs are CD133^+^, and when transplanted in immunodeficient non-obese diabetic (NOD)/severe combined Immunodeficiency (SCID) mice, they are able to develop tumor (29). Todaro and coworkers were the first to isolate TCSCs from primary thyroid tumors using ALDH activity. The authors highlighted the ability of TCSCs to form thyrospheres and recapitulate the parental tumor behavior when transplanted in murine thyroid gland. They found that a higher activity of ALDH in ATCs compared with FTCs and PTCs is correlated to the migration ability of TCSCs. ALDH^+^ TCSCs derived from ATC showed an enhanced migratory ability compared to ALDH^+^ cells derived from FTC and PTC. This phenotype is associated with an increase in c-MET and AKT activation. Silencing of these genes completely blocks the ALDH^+^ TCSCs metastatic capacity. Thus, c-MET and AKT have been proposed as potential therapeutic targets (20). Controversial data have been shown on cells derived from ATCs expressing stem cell markers, as Nanog and POU class 5 homeobox 1 (POU5F1) but lacking the expression of CD133 (30). Ahn et al. demonstrated that TCSCs present in PTCs express high levels of CD44, but no expression was revealed for CD24. The frequency of CD44^+^CD24^−^ CSC population was higher in recurrent PTCs than primary PTCs. POU5F1 was almost exclusively expressed by CD44^+^CD24^−^ cells compared with CD44^+^CD24^+^ cells (31). Shimamura et al. performed a comprehensive analysis of multiple markers (CD13, CD15, CD24, CD44, CD90, CD117, CD133, CD166, CD326, and ALDH activity) on eight cell lines derived from TCs and evaluated their ability to generate thyrospheres *in vitro* and tumor *in vivo*. This study identified ALDH activity and CD326 expression as reliable candidates to mark TCSCs (32).

Distinct signaling pathways are implicated in tumor initiation and progression by sustaining TCSC survival. In FTCs, the activation of mitogen-activated protein kinase (MAPK) and phosphatidylinositol 3-kinase (PI3K) pathways has been extensively described. In PTCs, the genetic alterations activate only MAPK pathway, while in ATCs, the MAPK, PI3K, and β- catenin pathways are activated. It has been widely demonstrated that *BRAF* and *RAS* mutations, *RET/PTC* rearrangements, and also *ALK* mutations activate MAPK pathway, which has a key role in thyroid tumorigenesis (33). The pathogenesis of TC, including angiogenesis, invasion, and metastasis process, is dictated by aberrant MAPK signaling pathway, altered production of chemokines, growth factors, matrix-metalloproteinases (MMPs), hypoxia-inducible factor 1α (HIF-1 α), and tissue inhibitor of metalloproteinases-1 (TIMP-1). In particular, in BRAF^V600E^ PTC, TIMP-1 is upregulated by nuclear factor kappa B (NF-κB) activation, leading to invasiveness, inhibition of apoptosis, increase in proliferation rate, and resistance to chemotherapy (34). Despite its role as MMP inhibitor, TIMP-1 exerts also its function in promoting tumor proliferation and regulating metastatic potential through hepatocyte growth factor (HGF) induced by HIF-1α. Thus, TIMP-1 could be used as a predictive biomarker and therapeutic target in advanced PTCs (35). PI3K-AKT pathway plays an important role in tumor progression and vascular intravasation in FTCs. In particular, elevated levels of AKT1 and its nuclear localization promote the invasiveness and the metastatic potential of FTC (36). This finding is supported by the presence of *AKT1* mutations in metastatic TCs (37).

Recently, it has been demonstrated that NF-κB regulates the proliferative and antiapoptotic signaling pathways in TCSCs. In ATC, the administration of NF-κB inhibitors in combination with radio- or chemotherapy exerts antiproliferative effects and induces massive apoptosis (38). Moreover, NF-κB is also upregulated by the MAPK pathway, suggesting a direct coupling with the presence of *BRAF*^*V*600*E*^ mutations as described above. In poorly differentiated TCs and ATCs, WNT-β-catenin signaling pathway is upregulated. This pathway has a role in the proliferation and differentiation of SCs, and its aberrant activation is a consequence of the activation of PI3K-AKT pathway by glycogen synthase kinase 3β (GSK3β) (39). Aberrant activation of the all above described signaling pathways represents the first step of TC tumorigenesis and can be considered as a target for novel therapeutic approaches.

## Epigenetic Alterations Sustaining TCSCs Origin and Maintenaince

**Figure 2:**
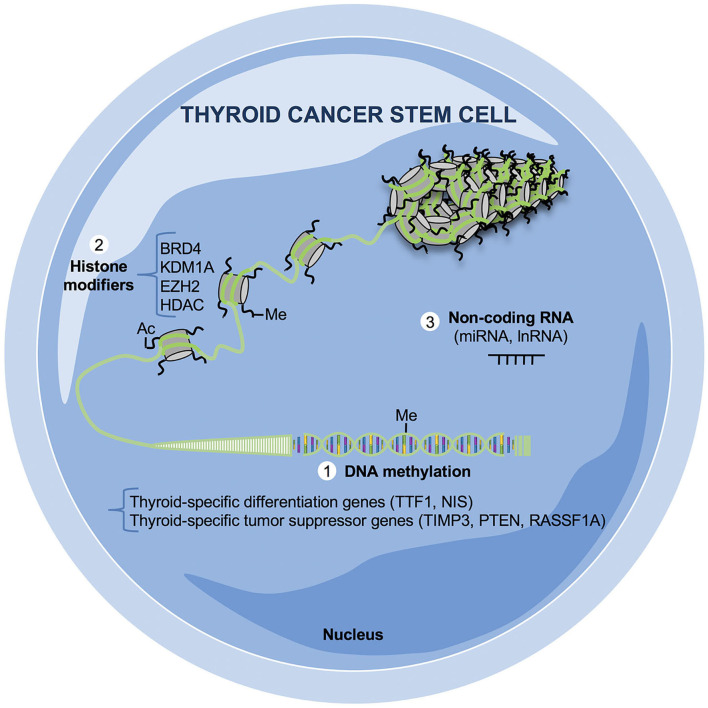
Epigenetic mechanisms relevant in thyroid cancer stem cells (TCSCs). Altered epigenetic mechanisms crucial for the survival of TC cells and, in particular for TCSCs, have not completely elucidated yet. The majority of aberrant epigenetic events in TCs are based on (1) DNA methylation: the promoters of thyroid-specific differentiation as transcriptional thyroid factor 1 (*TTF1*) and sodium iodide symporter (*NIS*) and of tumor suppressor genes as tissue inhibitor of metalloproteinase-3 (*TIMP3*), phosphatase and tensin homolog (*PTEN*), Ras association domain family 1 isoform A (*RASSF1A)* are silenced by hypermethylation; (2) histone modifications: several histone-modifying enzymes are aberrantly expressed in TCs as bromodomain-containing protein 4 (BRD4), lysine demethylase 1A (KDM1A), enhancer of zeste homolog 2 (EZH2), and histone deacetylase (HDAC); (3) non-coding RNA (miRNAs, lnRNAs): many non-coding RNAs are up- or downregulated in both well-differentiated and undifferentiated TCs, influencing the transcription and the posttrancriptional regulation of several oncogenes and/or tumor suppressor genes.

Epigenetic regulation of gene expression and chromatin compactness is a reversible and dynamic process crucial in developmental biology and in many diseases, including cancer (40). The impaired differentiation of normal stem cells into tissue subtypes and their acquisition of CSC capabilities as self-renewal, chemoresistance, and metastatic potential are finely governed by epigenetic modulators (9). Epigenetic alterations fundamental in the tumorigenesis of many cancer types, including TC, are mainly related to the following mechanisms and/or modulators summarized in Figure 2: (1) DNA methylation, inducing the silencing of many tumor suppressor gene transcription through the DNA methyltransferases (DNMTs); (2) histone modifiers, which regulate gene expression by adding or removing mostly acetyl or methyl groups to the histone tails; (3) Chromatin remodelers that control the structural organization of chromatin by modulating its degree of condensation; (4) non-coding RNAs, including microRNAs (miRNAs) and long non-coding RNAs (lnRNAs), which may exert their role in both inhibiting or aberrantly inducing gene transcription. In thyroid tumors many epigenetic modifiers are altered, but further studies are needed to better elucidate how the complex regulation of histone modifying enzymes and chromatin remodelers could interfere with or facilitate the tumor growth of the CSC subset. Here, we highlight the key points related to the most relevant epigenetic mechanisms altered in the different subtypes of TCs: (i) Thyroid-specific differentiation and tumor suppressor genes aberrantly methylated in their promoter regions along with altered expression of miRNAs represent the most frequent epigenetic features in well-differentiated thyroid tumors, as PTC and FTC, while the amount of reports regarding the histone modifications are very limited. (ii) The most common altered epigenetic mechanisms that contribute to well-differentiated TC initiation and progression, lead to the activation of MAPK and PI3K/AKT survival pathways, which sustain TCSCs origin and maintenance. (iii) In UTCs in addition to MAPK and PI3K/AKT pathways, other signaling pathways, as Wnt/β-catenin and Notch, crucial for the survival of TCSCs are epigenetically compromised. Unlike the well-differentiated thyroid tumors, the DNA methylation profiling in the undifferentiated subtypes reveals more often the aberrant promoter hypomethylation compared with hypermethylation.

### DNA Methylation in Thyroid Tumors

Global DNA methylation studies utilizing next generation sequencing (NGS) platforms (41–43) have identified different DNA methylation patterns associated with TC histological variants and their distinct mutational status. Specifically, the hypermethylation has been associated with the well-differentiated TCs while the hypomethylation with the undifferentiated ones. DNA methylation status has been correlated with *BRAF* mutation, tumor progression, and aggressive behavior in PTCs (44). Thus, genome-wide approach shed new lights on the methylome profiles of TC subtypes; nevertheless, novel potential biomarkers for early diagnosis, prognosis, and therapy resistance have not been identified yet. The majority of the thyroid-specific tumor suppressor genes, which have been found aberrantly methylated in their promoter regions, exert their role as antagonists or modulators of PI3K pathway, RAS signaling, and EMT process. Of note, *PTEN* (phosphatase and tensin homolog) acts as an inhibitor of PI3K pathway (45); RAS association domain family 1A *(RASSF1A*) (46) and *RASAL* (RAS protein activator like-1) (47) are both involved in RAS signaling, cell cycle regulation, and mitotic progression; *TIMP3* (tissue inhibitor of metalloproteinase 3), is an inhibitor of metastasis, angiogenesis, and invasion; and others are known as tumor suppressors as the death associated protein kinase *(DAPK)* and the retinoic acid receptor beta2 *(RAR*β*2)* (44). Silencing of E-cadherin (*CDH1*) by its promoter methylation has been hypothesized as a potential mechanism of enhanced EMT in FTC cells, whereas in PTC cells, *CDH1* expression levels are maintained (48, 49). Moreover, thyroid differentiation specific genes, as thyroid transcription factor-1 (*TTF1*) and sodium-iodide symporter (*NIS)*, are also hypermethylated particularly in undifferentiated tumors (50). High expression levels of DNMT1 have been associated with silencing of *NIS* (51). The role of KISS1/KISS1R signaling is controversial in various types of cancers, as it has been associated with both roles in metastatic promotion and suppression (52–54). Savvidis et al. found increased levels of KISS1 in extrathyroidal tissues of advanced differentiated TCs while an inverse correlation between KISS1R and tumor size (55). Interestingly, it has been demonstrated that *KISS1R* promoter is hypermethylated particularly in FTCs (42). Therefore, further studies are needed to define the methylome profile in TCSC population in an attempt to find specific targets in this cell compartment. However, the fact that components of the principal pathways involved in TCSC survival are silenced by hypermethylation strongly suggests that this could be a mechanism specific also of this subset.

### Histone Modifications and miRNAs Role in Thyroid Tumors

Aberrant alterations in the histone modifier epigenetic enzymes as well as in the chromatin remodeling complexes have not been completely studied in TCs and in the subset of TCSCs. Nowadays, there are few reports about the histone modifications in TCs. Histone deacetylase (HDAC) enzymes modulate the levels of histone tail residues acetylated. It is now widely accepted that treatment with HDAC inhibitors is very effective in TCs subtypes, as they impair cell growth and induce apoptosis of TC cells while increasing the radioiodine uptake. Of note, the global acetylation levels in TCs are higher than in healthy tissues (56). Moreover, UTCs express lower levels of acetylated H3 on lysine 18 compared with well-differentiated TCs, suggesting that the deacetylation is a step required for the acquisition of a more aggressive phenotype. Interestingly, in cells with reduced levels of *TTF1* because of hypermethylation, levels of acetyl H3K9 are reduced and dimethyl H3K9 are increased. BRD4 is a bromodomain protein, which binds the acetylated histones facilitating the recruitment of transcription factors and ultimately the transcription. The role of BRD4 as a potential oncogene in TCs has been recently elucidated. Levels of BRD4 are higher in ATCs compared to healthy tissues. The BRD4 inhibition, by JQ1, which also inhibits c-Myc and induce *TTF1*, or by AZD5153, impairs TC cell growth, suggesting that it is critical for thyroid proliferation (57, 58). KDM1A is a H3K9 demethylase frequently overexpressed in PTCs and required for migratory and invasive capabilities of PTC cells (59). TIMP1 has been described as a KDM1A target. KDM1A epigenetically silences TIMP1 and subsequently activates MMP9 promoting metastasis and migration (59). Finally, the enzymatically active component of the PRC complex, EZH2, involved in the regulation of embryonic development and responsible for the trimethylation of lysine 27 on H3 (H3K27me3), leading to silencing of gene transcription, is overexpressed in ATCs (60). Future studies are needed to better characterize the epigenetic modifications and their use as potential therapeutic targets in TCs.

miRNAs are short molecules of 19–23 nucleotides involved in blocking transcription or degradation of messenger RNA (mRNA), which have been associated with critical roles in TC initiation and progression because they can downregulate tumor suppressor genes or upregulate oncogene transcription. Their expression is also specific for each TC subtype. An extensive overview about the principal aberrant epigenetic events and, in particular, the most differentially up- or downregulated miRNAs involved in both well-differentiated or undifferentiated TCs is reported in (61, 62).

### Epigenetic Therapy in Thyroid Tumors

Epigenetic drugs can be used to revert the chemoresistance of TCs. Given that epigenetic modifications are reversible, these may be approached differently than genetic mutations, which are irreversible. HDAC and DNMT inhibitors represent the first Food and Drugs (FDA)-approved epigenetic drugs and are currently used in several clinical trials showing promising results for TC treatment (63). Recently, many *in vitro* and *in vivo* studies have reported that treatment with HDAC inhibitors alone or in combination with chemotherapy or other biological agent induces apoptosis and cell cycle arrest, impairing the growth while incrementing the radioiodine uptake of TC cells (64). Moreover, HDAC inhibitors, as Trichostatin A (TSA), and Valproic acid (VA) combined with DNMT inhibitors as azacitidine, exerts an antitumoral effect by reducing MMP2 and MMP9 levels in PTC and FTC. Unfortunately, some clinical limitations have been associated with the use of the epigenetic inhibitors in many solid tumors, including TC, as remethylation, the lack of specificity and general toxicity. Although the use of DNMT inhibitors was expected to revert the silencing of many tumor suppressor genes involved in TC tumorigenesis, it has been demonstrated that they also promote oncogenes and prometastatic genes transcriptional activation through a global change in gene expression. Currently, only two clinical studies verified the efficacy of demethylating agents in patients affected by recurrent and/or metastatic differentiated TCs, with no partial or complete responses and severe side effects (www.clinicaltrials.gov, NCT00085293 and NCT00004062) (65). Nevertheless, these inhibitors may be potentially translated in clinical settings in combination with HDAC inhibitors and/or other targeted therapies such as mammalian target of rapamycin (mTOR) inhibitors (66). Notably, HDAC inhibitors, although generally well-tolerated, induced cardiac arrhythmias in patients with heart disease. The final data from the ongoing clinical trials are needed to understand their efficacy in treating TC refractory to current therapies (67, 68). The hurdles in the administration of these epigenetic drugs will be overcome with the use of highly specific inhibitors (for instance HDAC inhibitors specific for a single class or a single HDAC) and/ or nanoparticle (liposomes, nanogels, polymeric nanoparticles) to reduce off-target effects and improve the delivery system (69). Overall, so far, the successful preclinical results obtained in TC cells treated with the epigenetic drugs have not been recapitulated in clinical settings mostly due to the epigenetic landscape heterogeneity of thyroid tumors, the influence of immune cells, and the lack of a complete knowledge about the other TC histological subtypes other than PTCs. A better understanding of the epigenetic changes in TCSCs is crucial to design more effective molecular therapies (70).

## The Cross-Talk Between Immune System and CSCs in Thyroid Cancer

The first barrier to tumorigenesis is the immune surveillance. CSCs represent a heterogeneous subpopulation able to modulate the host immune response and ultimately escape the attacks mediated by the immune cells. The experts define this phenomenon as cancer immunoediting, a process that includes three different phases: the elimination, the equilibrium, and the escape. The elimination phase consists in the ability of innate and adaptive immune cells to recognize and destroy CSCs (71). In the equilibrium phase, the tumor is not eliminated but contained. In this phase, the immune system eliminates the immunogenic cancer cell clones. In the escape phase, the quiescent state and the low immunogenicity, typical of CSCs, allow them to remain in their niches without being recognized from host immune system. In particular, CSCs can escape immune destruction by reducing their expression of major histocompatibility complex I (MHC I) and by completely eliminating the expression of MHC II and co-stimulatory molecules.

The CSCs can also minimize the host immune response recruiting immunosuppressive cells by secretion of growth factors and interleukins, such as transforming growth factor beta (TGF-β), interleukin (IL)-6, IL-10, and prostaglandin E_2_ (PGE2) (72). Immune cells with pro- and antitumorigenic roles have been detected in thyroid TME (73). Of note, specific classes of immune cells can be related to TC patient outcome. Unlike the infiltration of natural killer (NK) cells, presence of T regulatory lymphocytes in PTC is positively associated with advanced disease (74). Currently, in TC TME, the role of tumor-associated macrophages (TAMs), tumor-associated neutrophils (TANs), and tumor-associated mast cells (TAMCs) has been extensively described, while few studies have been reported regarding the presence of NKT cells, γδ T cells, Th9, and Th17 in different TCs (75–77).

**Figure 3:**
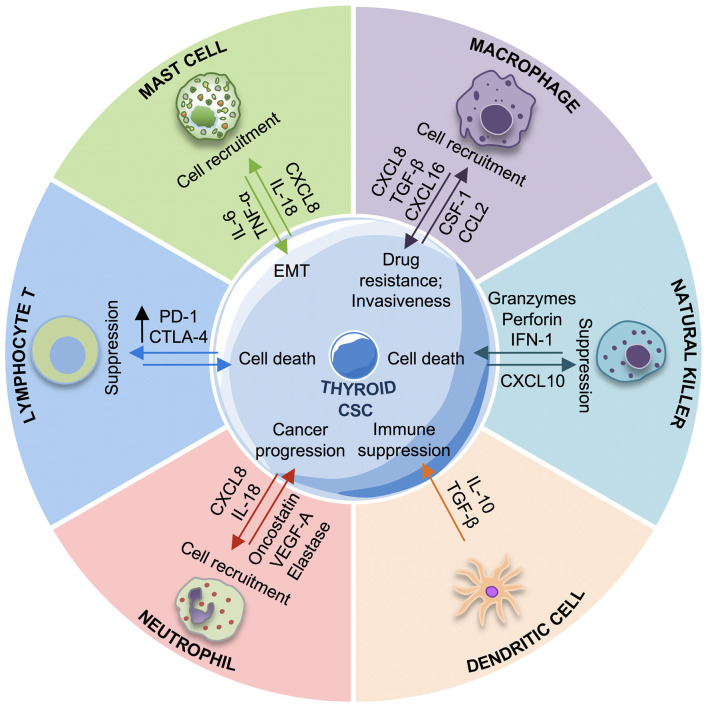
Cross-talk between immune system and thyroid cancer stem cells (TCSCs). The schematic picture describes the interactions between TCSCs and different innate and adaptive immune cells. TCSCs escape from immune response by different mechanisms: recruiting macrophages M2, mast cells, neutrophils with protumorigenic roles promoting the immunosuppressive response through the release of interleukin (IL)-10 by MDSCs and iDCs, suppressing the NK cells response by PGE2, and upregulating CTLA-4 and PD-1. Conversely, the immune response contrasts the TCSC growth and proliferation by CD8+ T lymphocyte and NK cells cytotoxic activity. EMT, epithelial-to-mesenchymal transition; iDC, immature dendritic cells; IL-6, interleukin-6; IL-10, interleukin-10; CXCL-8, C–X–C motif chemokine ligand-8; CXCL16, C–X–C motif chemokine ligand 16; TGF-β, tumor growth factor-β; VEGF-A, vascular endothelial growth factor A; TNF-α, tumor necrosis factor-α; PGE2, prostaglandin-E2; CXCR3, CXC chemokine receptor; CCL-2, C–C motif chemokine ligand-2; csf-1, colony stimulating factor-1; NK cells, natural killer cells.

In the following paragraphs, we will review in details the bidirectional cross-talk between the TCSCs and immune cells (Figure 3).

### Immune Cells and Their Protumorigenic Role

The innate and adaptive immune cells are in charge of the defense against foreign agents. Among the immune cells, NK cells are the first to recognize and kill CSCs, with the resulting apoptotic cell fragments being eliminated by macrophages. Tumor neoantigens are processed and presented to the T cells by dendritic cells (DCs). The release of cytokines by DCs and other immune cells induce the activation of T and B cells promoting an inflammatory environment that further stimulates the innate immunity and supports the expansion of T cells and the production of antibodies toward antigens expressed on CSCs. In TME, immune cells exhibit not only an antitumorigenic role, but they can also induce a protumorigenic and anti-inflammatory condition contributing to CSC subpopulation survival (71). In TC TME, TAMs can exert different functions by dynamically interchanging between M1 (proinflammatory) and M2 (anti-inflammatory) phenotypes (78). TAMs polarize into M1 phenotype in presence of interferon gamma (IFNγ) and lipopolysaccharide (LPS), while acquiring an M2 phenotype for IL-4 and IL-13 action. Typically, TAMs with M2 phenotype play an important role in promoting tumor angiogenesis and progression (79) and are correlated with poor prognosis in several tumors, including TCs (80). In the different subtypes of TCs, the frequency of TAMs is variable and has been correlated with various prognoses. In ATCs, TAMs represent 50% of all immune cells and correlate with poor prognosis (81). In PTCs, the presence of TAMs is associated with large size tumors, presence of lymph node metastasis, and decreased survival (80, 82, 83). In poorly differentiated TCs, TAMS are correlated with negative outcomes as capsular invasion and extrathyroid tumor extension (80). The interaction between TAMs and TCSCs can be directly and indirectly mediated by the release of many cytokines and chemokines. It has been demonstrated that TAM-secreted CXCL8 and CXCL16 can promote the metastatic process in PTCs. Blocking CXCL8 and CXCL16 signaling reduced the invasiveness of PTC cells (83, 84), suggesting their targeting as a possible therapeutic strategy in PTCs. In PTCs, TGF-β produced by TAMs is overexpressed and correlates with the presence of CD68^+^ TAM infiltration and with tumor invasion (85). In BRAF-induced PTC mouse model, Ryder et al. observed an increased of expression of two TAM chemoattractants: colony stimulating factor-1 (Csf-1) and CCL-2. The increase in Csf-1 is correlated with a high number of infiltrating TAMs expressing Csf-1r and CCR2 and with PTC progression (86). A high number myeloid derived suppressor cells (MDSCs), considered as precursors of monocytes and neutrophils, has been reported in cancer patients as index of poor prognosis. In differentiated TCs, the presence of MDSCs has been correlated with tumor aggressiveness (87). MDSCs can be identified as M-MDSCs or PMN-MDSCs, with a phenotype similar to monocytes or neutrophils, respectively. Both cell types are activated in the TC microenvironment by CCL2, CCL5, and CSF-1 and act as inhibitors of the antitumor response by releasing IL-10 (88). Many studies have suggested that chemotherapy or other treatments such as tyrosine kinase and nitric oxide inhibitors improve TC patient prognosis by inducing MDSC differentiation or by inhibiting their function (89). Unlike other immune cells, DCs are not usually present in TCs, except for PTCs bearing *BRAF*^*V*600*E*^ mutation. In PTCs, DCs are immature cells (CD1a^+^ and S100^+^) able to secrete cytokines as IL-10 and TGF-β, thereby promoting immune suppressive response (90). Similarly to DCs, Tregs are also elevated in PTCs and contribute to immune suppressive TME, favoring tumor cell survival. The mechanisms underlying this function have not been completely clarified; nonetheless, it has been hypothesized that Tregs induce an anergy state in T effector cells, which become unable to recognize and attack the CSCs (74).

Mast cells are the first immune cells recruited during the inflammatory process, and they can also exert a protumorigenic role (91). The presence of mast cells has been associated with extrathyroidal tumor extension in 95% of PTCs and with invasiveness in poorly differentiated TCs and ATCs (77). Tumor-associated mast cells are more abundant in thyroid follicles of patients affected by PTCs compared with patients with thyroid adenoma (92). By releasing IL-6, TNF-α, and CXCL8/IL-8, mast cells can promote the EMT process in thyroid cells derived from all TC subtypes (FTC, ATC, and PTC). To date, the role of neutrophils in cancer is controversial. An elevated number of neutrophils in cancer patients have been correlated with better clinical outcomes (93–95) or with tumor progression (96, 97). TCSCs recruit the neutrophils by releasing CXCL-8/IL8 and upregulate their proinflammatory roles, thus promoting tumor progression. Galdiero et al. in TCs showed a correlation between the density of neutrophils and the tumor size, suggesting that these immune cells are involved in the tumorigenesis process. It has been demonstrated that TANS favor the tumor proliferation and invasion by the secretion of cytokines as oncostatin M and vascular endothelial growth factor A (VEGF-A) and by elastase action (98). In TC patients, it has been observed that a higher neutrophil/lymphocyte ratio correspond to a larger tumor size and high risk of recurrence (76). NK cells are responsible for the earliest stages of immune defense against microbe infections or the expansion of stressed or transformed cells. NK cells possess a cytotoxic activity, and their activation depend on two factors: they recognize the stimulatory receptors in the target cells and simultaneously their inhibitor receptors fail to link MHC I molecules on foreign cells. It is possible to distinguish two populations of NK cells, with different localization and functions: the CD56^bright^/CD16^dim^ cells, which predominate in the circulation and the CD56^dim^/CD16^bright^ cells, which are more cytotoxic and present in the majority of tissues (99). In the tumor, the NK cells CD56 ^bright^ have a key role to eliminate the nascent tumor cells, identified as stressed and abnormal cells, with a low expression or lack of expression of MHC I class. In TCs, the role of NK cells needs further investigations. In PTC patients, the presence of NK cells is negatively correlated with disease stage (100). In fact, some studies have demonstrated that ATC cell lines are susceptible to NK-mediated immunotherapy (101, 102). However, tumor immunosuppression by NK targeting may also represent an obstacle to the activation of their cytotoxic function (103, 104).

### TCSCs and Evasion Mechanisms

CSCs can attenuate the immune surveillance contributing to the tumor development. CSC interaction with immune cells consists in the secretion of immunosuppressive factors or the recruitment of immunosuppressive cells. In particular, CSCs downregulate some key elements for the antigen processing and presentation, contributing to their immune privileged status, thus leading to the inactivation of T cells (105). CSCs can escape NK cell action by different mechanisms: (i) increased expression of ligands that bind the inhibitor NK receptors; (ii) recruitment of T regs, which can promote the immune evasive state; (iii) NK and T-cells-mediated IL-22 secretion, which promotes CSC phenotype (72). The activation of a T-cell-mediated immune response needs not only the presence of MHC molecules but also of co-stimulatory molecules as CD80 and CD86. Tumor cells downregulate CD80/CD86 expression while upregulates the expression of PD-L1 (B7-H1), which binds to PD-1 on T cells promoting their anergy state (106). Thus, targeting PD-L1 represents a promising immunotherapy approach in many cancers and in advanced differentiated TCs and ATCs (107).

### Immunotherapy and TCSCs

CSCs are resistant to different chemotherapeutic agents and radiotherapy due to high expression levels of drug efflux pumps, efficient DNA repair mechanisms, and the support of TME. Immunotherapy approach has been developed as a novel frontier also in targeting CSCs (108). Cancer immunotherapy consists in the administration of recombinant antibodies toward immune molecules defined as immune checkpoint (PD-1, CTLA-4), along with recombinant cytokines, oncolytic viruses, cancer vaccines, or engineered T cells (109). Given that immune cells play a key role in supporting the initiation and progression of TC, the identification of specific immune targets could improve the efficacy of TC therapy. Different immunotherapy strategies have been implemented for the treatment of TC: (i) failure of TAM recruitment, (ii) TAM polarization in M1 phenotype, (iii) identification of tumor antigens for the development of cancer vaccines, and (iv) inhibition of immune checkpoints (110). TAMs are highly represented in ATC TME, as compared to advanced differentiated TCs. High levels of CCL-2 and CSF-1, two TAMs chemoattractants, have been found in human TC tissues (82). In this context, blocking and targeting CCL-2/CCR-2 and CSF-1/CSF1R inhibits the recruitment of TAM with M2 phenotype and the repolarization of these cells into the anti-tumor M1 phenotype (86, 111). To date, clinical trials are ongoing to test the efficacy of the CSF-1R antibody (LY3022855) and CSF-1R inhibitor (PLX3397) (NCT01346358 and NCT01525601, respectively). TAMs represent the half of TME cells in ATC; in this way, the block of CSF-1 and CCL-2 represent a potential target therapy. TC cells express a specific inhibitory receptor membrane, CD47 that binds SIRPα, a ligand expressed on TAMs. The interaction receptor–ligand leads to inhibition of phagocytosis by TAMs and affects the tumor antigen presentation by DCs by supporting TC progression. The block of SIRPα/CD47 interaction in mouse model leads to tumor growth impairment and regression. Use of a specific antibody represents a way through which this pathological pathway re-educated in TAMs (112). Specifically, *in vitro* study using the anti-CD47 on TC cell lines shows the induction of apoptosis (113). Another potential strategy could identify TC-specific antigens to develop a successful vaccine for T cells and dendritic cells in the context of TME. Some studies suggest that potential TC antigens, as the proto-oncogene c-MET, melanoma antigen encoding genes (MAGE), and mucin-1 antigen (MUC-1), are co-expressed with thyroglobulin and thyroid peroxidase in differentiated TCs (114, 115). A promising study demonstrated that dendritic cells vaccines targeting the carcinoembryonic antigen (CEA), an antigen highly revealed in MTC, induced a complete regression of metastases in lung and liver (116). Further clinical studies are ongoing to test adoptive cytotoxic T cells that target preferentially expressed antigen in melanoma (PRAME), New York esophageal squamous cell carcinoma 1 (NY-ESO-1), MAGE Family member A4 (MAGEA4), synovial sarcoma translocated gene (SSX), and survivin in patients with advanced tumors, including TCs. Currently, the immune checkpoints (PD-1, CTLA-4) inhibition represents a valid strategy for treatment of different types of cancers. Blocking these pathways strengthens T cells and inhibits T reg suppressor cells. In TC histological subtypes, different levels of PD-L1 expression have been detected (7.6% of FTC, in 6.1% of PTCs, and 22.2% of ATCs) (117). Moreover, PDL-1 has been found expressed in more than the half of 126 cases of primary PTCs, and its expression positively correlates with rich tumor-infiltrating lymphocytes (118). Two antibodies targeting PD-1, the receptor of PD-L1 (Pembrolizumab and Nivolumab), have been FDA-approved for treatment of melanoma, renal carcinoma, and non-small cell lung carcinoma, and recently also for PTC patients. Specifically, clinical trials are ongoing to test the efficacy of Pembrolizumab in association with PLX3397 in TCs to better maximize the clinical results in these tumors (NCT02452424). Although the immunotherapy needs further evaluation in TCSC compartment, it may represent a reliable strategy for TC treatment.

## Influence of the Tumor Microenvironment on TCSCs

**Figure 4:**
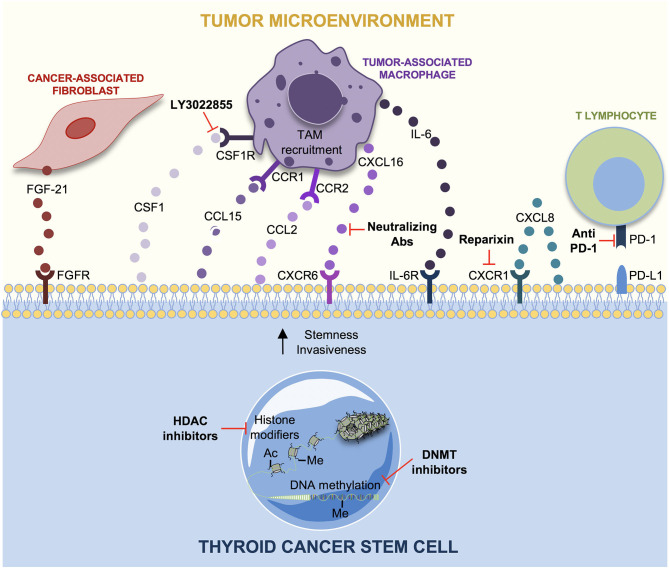
Tumor microenvironment (TME) interactions and therapeutic strategies in thyroid cancer stem cells (TCSCs). The principal interactions between TCSCs and tumor microenvironment (TME) cells, along with the novel potential therapeutic targets, are shown. Cancer-associated fibroblasts (CAFs) produce FGF-21, which favors a more aggressive TC cells phenotype. TC cells secrete chemokines as CSF1, CCL15, and CCL2 to recruit tumor-associated macrophage (TAM) in order to sustain TC progression. CXCL8 secreted by TC cells guarantees stemness through an auto-paracrine loop by binding CXCR1. CXCL16 is secreted by TAMs favoring the migration and invasiveness of TC cells. Interleukin-6 (IL-6) supports stemness, colony formation and proliferation, in TC cells through JAK1/STAT3 phosphorylation. Reparixin (CXCR1 inhibitor) decreases thyrospheroid survival and epithelial–mesenchymal transition (EMT). The use of the CSFR1 inhibitor, LY3022855, hampers TAM recruitment. CXCL16-neutralizing antibody counteracts TC progression. Immunotherapy is based on the use of anti-PD1 antibodies as Pembrolizumab and Nivolumab, whereas epigenetic therapy nowadays is limited to histone deacetylase (HDAC) and DNA methyltransferase (DNMT) inhibitors alone or in combination with conventional approaches in TCs.

TME is composed of different cell types (endothelial cells, fibroblasts, immune cells) and extracellular components (cytokines, chemokines, exosomes, growth factors, hormones, extracellular matrix) that support tumor growth. TME exerts an important role not only during tumor initiation, progression, and metastasis but also in drug resistance. Therapy refractoriness is mediated by a continuous crosstalk between tumor and stromal cells (119, 120). In TCs, an inflammatory TME has been involved in thyroid carcinogenesis. Specifically, the molecular interplay between cytokines and chemokines with a protumorigenic role could explain how the inflammation could favor TC initiation (121). TCSCs survival is crucially modulated by TME cells, through a bidirectional complex network of chemokines/cytokines and the induction of an EMT-like phenotype mediated by tumor-associated fibroblasts (CAFs) and by exosomes release (Figure 4).

### TCSCs and the Interplay With Chemokines/Cytokines

The interplay between TCSCs and stromal cells supports tumor progression by recruiting fibroblasts or macrophages to facilitate metastasis. This crosstalk is mediated by different kinds of chemokines, which are low molecular weight peptides with proangiogenic, cytoproliferative, and prometastatic properties (122). Among the main players of thyroid tumorigenesis, many chemokines and cytokines have been proposed such as CXCL8, CCL2, CXCL16, CCL15, and IL-6 (123). Of note, FTC cells produce CCL15, which is a chemokine that binds CCR1 receptor expressed by TAMs to facilitate their recruitment and to favor tumor progression and metastasis (124). Studies of co-culture of macrophages and PTC cells showed that CXCL16, abundantly secreted by TAMs, promotes PTC cells migration and invasiveness. It has been demonstrated that TC progression is arrested by using an antihuman CXCL16 neutralizing antibody (84). CCL2, a chemokine secreted by PTC cells, facilitates TAM recruitment in thyroid TME. CCL2 production has been positively correlated with *BRAF*^*v*600*E*^ mutations in PTC cells and with TAM infiltration (123). Several studies show how CXCL8, a chemokine secreted by TC cells, plays a protumorigenic activity by enhancing mechanisms such as stemness and EMT in TC cells. In fact, *in vitro* data showed that CXCL8, through an auto-paracrine circuit, binds CXCR1 receptor and induces the tumor sphere formation, typical of CSCs (77). CXCL8's role in carcinogenesis is also confirmed by *in vivo* studies that show as the inoculation of TC cell line, BCPAP, in NOD/SCID mouse model induces metastasis if treated with exogenous recombinant human CXCL8 (83). TAM-derived IL-6 has a pivotal role in tumor initiating, survival, proliferation, and drug resistance in solid tumors (125–128). Similarly, IL-6 drives TC progression even if its molecular mechanism is unknown (129, 130). Zheng et al. studied the effect of IL-6 on ATC-derived CSCs showing that the *in vitro* treatment of HTh74 and HTh74R TC cell lines with exogenous IL-6 leads to increased number of tumor spheres and stemness markers, OCT4 and CD133. IL-6 promotes the colony formation and EMT through the upregulation of vimentin and Snail and the downregulation of E-cadherin in HTh74 and HTh74R TSCSs, contributing to TC progression. In addition, IL-6 increases dramatically TSCS growth and proliferation through JAK1/STAT3 pathway activation (131).

### TCSCs and Tumor-Associated Fibroblasts (CAFs)

Cancer cells establish a complex relationship with the surrounding stromal cells such as cancer-associated fibroblasts (CAFs). During tumor progression, CAFs create a “reactive stroma” by releasing a series of signals inducing phenotypic changes in cancer cells, which reciprocally communicate with CAFs (132). Many studies have shown a positive correlation between specific mutations in TC cells and fibroblast activation. For instance, *BRAF*^*V*600*E*^ mutation in TC cells has been involved with a metastatic phenotype by modulating TME and the extracellular matrix (ECM) (133). *BRAF*^*V*600*E*^ mutation is associated with upregulation of integrin and fibronectin, which compose the ECM. The upregulation of ECM-associated genes induces the formation of a fibrotic tumor stroma in which an increased number of fibroblasts is recruited to facilitate TC progression (134). Further studies showed that several ATC cell lines are able to reprogram human normal fibroblasts in CAFs to support tumor growth. Treatment of quiescent fibroblasts with conditional medium from ATC cells induces their transformation in CAFs. These findings have been confirmed by the evaluation of fibroblast-specific markers such as platelet-derived growth factor receptor α (PDGFR-α) and alpha smooth muscle actin (α-SMA). Therefore, the activated CAFs acquire a more intense metabolic and proliferative activity and a secretory phenotype, which improves the invasiveness and aggressiveness of FTC cells (135). Taken together, these findings demonstrated that factors secreted by CAFs, upon activation by ATC cell-derived conditioned media (CM), represent key modulators in TC progression. Fibroblasts are able to secrete fibroblast growth factor 21 (FGF-21) and to release it into blood circulation by acting at distance on TC cells. High FGF-21 levels increase EMT in PTC through FGFR signaling pathway. Treatment of PTC cell lines with recombinant FGFR1 leads to FGFR pathway signaling activation, leading to ERK and AKT phosphorylation, EMT, and to the development of a more aggressive phenotype of tumor cells. Thus, FGFR1 can promote invasion and migration of PTC cells (136). Parascandolo et al. showed that mesenchymal stem cell (MSC) cultures derived from non-tumorigenic thyroid tissues and from PTCs, exposed to cancer cell-released factors, are able to differentiate into a multitude of cell types, including CAFs, which eventually support tumor progression. Of note, a subpopulation of PTC cell line, expressing the TCP1 marker, influences the switch from autocrine to paracrine MSC-mediated secretion of superoxide dismutase 3 (SOD3). This leads to MSC activation and differentiation, with the upregulation of fibrotic markers such as FAP, tenascin and Col1 A1, suggesting the presence of CAFs in TC microenvironment. Stromal SOD3 exerts a stimulatory effect on TC cell growth representing a potential target for the treatment of these tumors (137). TIMP family is characterized by a specific structure, with the C-terminal region able to bind specific parts of MMPs in order to form MMP–TIMP complex, which inhibits tumor metastasis and invasion. However, TIMP family components as TIMP-1 and TIMP-2 could play opposite functions as promoting tumor growth, inhibiting apoptosis, and contributing to therapy resistance in different types of cancer, including PTCs (138–140). Consistently, TIMP-1 expression has been detected in fibroblasts of differentiated thyroid carcinoma and thyroid adenoma. Its expression levels are positively correlated to tumor growth, tumor–node–metastasis (TNM) stage, metastasis, and recurrence. In particular, Zhang et al. found that the presurgery peripheral blood levels of TIMP-1 and MMP9 in patients with differentiated TCs are significantly elevated, although TIMP-1 expression levels still remain lower than MMP-9 levels, thus promoting the development of cancer because the balance between the two was broken (141).

### TCSCs and Exososomes

Exosomes are a subtype of extracellular vesicles, which originate in the endosomal cell compartment via multivesicular bodies (MVBs). Upon their maturation, exosomes emerge from the extracellular membrane and initiate a cross-talk with bulk tumor cells (142). Cancer cells and cancer-associated cells in TME, as fibroblasts and macrophages, release exosomes through which they transfer specific molecular messages between malignant and non-malignant cells and activate pathways that facilitate tumor initiation and progression (143). Specifically exosomes mediate intercellular communication by the so-called “exosomal cargo,” which includes proteins, miRNAs, and mRNAs (144). In TC, exosomes derived from TCSCs have a central role in supporting metastasis by enhancing EMT through non-coding RNA transfer. Hardin et al. used a subpopulation of the TCP1 TC cell line, expressing adult stemness markers, and isolated exosomes from them. Treatment with exosomes of normal thyroid cell lines NTHY-ori-3 leads to the upregulation of long non-coding RNAs (lncRNAs) (MALAT1 and linc-ROR), whose molecular function is unclear, of the EMT marker SLUG and the stem cell marker SOX2 in normal cells, which acquire and increase their proliferative ability. TCP1-derived exosomes are able to activate EMT program in the same cells contributing to a more aggressive phenotype (145).

### TCSCs and EMT

EMT is a clear example of cellular plasticity, through which epithelial cells develop a mesenchymal phenotype that enhances their migratory and invasive features (146). Different studies underlined the correlation between EMT process and an increased number of TCSCs in TCs (145, 147, 148). Consistently, Yasui et al. by analyzing thyroidectomy specimens, found that ATC regions coexisted with DTC. ATC regions showed absence of E-cadherin and a dramatic upregulation of the stem cell markers CD44, CD133, and of a neuronal marker nestin. Meanwhile, differentiated TCs and non-neoplastic regions showed a decrease in stem markers expression and nestin, but they were positive for E-cadherin (149). Furthermore, Heiden et al. demonstrated that sonic hedgehog pathway promotes the maintenance of a CSCs pool in ATC. They observed that Gli 1-induced Snail upregulation and increase ALDH^+^ CSCs number in ATC cell lines (150). Hardin et al. reported that PTC cell lines acquired stem-cell-like features simultaneously to TGF-β-induced EMT. Interestingly, PTC cells upregulated a novel EMT activator paired-related homeobox protein 1 (Prrx1) (151). Ma et al. confirmed the upregulation of SSEA1 in TSCSs, which were positive also for Nanog, Sox2, and Oct4. They found a correlation between stemness and EMT; indeed, TSCSs showed EMT initiation through the increased expression of vimentin and snail and decreased expression of E-cadherin (152). Mato et al. showed for the first time the correlation between the expression of stem markers ABCG2 and the expression of EMT activator genes. They identified the subpopulation of PTC cells named PTC1, expressing ABCG2. PTC1 cells were characterized by an aggressive phenotype and showed high propensity to migration, invasion, and apoptosis inhibition due to *BIRC5* gene expression. For these reasons, PTC1 could have a pivotal role in PTC progression (153). To overcome the lack of nutrients and oxygen within the tumor mass, tumor cells foster the formation of newly synthesized vessels endowed with irregular structure and properties. Recently, it has been demonstrated that patient-derived xenografts of thyroid spheroids obtained from PTCs show “stem-like” features and promote neoangiogenesis in zebrafish *in vivo* model (154). Interestingly, Cirello et al. proposed an additional experimental model resembling the original thyroid tissue, composed of thyroid stem-like cells and endothelial and hematopoietic cells (155). These 3D models allowed to evaluate the therapeutic response to antiangiogenic compounds and other anticancer drugs and to assess the impact of several proangiogenic factors, as VEGF, FGF, and TSH, in TC progression (155, 156). Moreover, also pericytes can regulate the interaction between tumor and endothelial cells. Specifically, pericytes protect tumor cells by releasing proangiogenic factors. In TCs, it has been demonstrated that pericytes confer resistance to *BRAF*^*V*600*E*^ inhibitors and tyrosine-kinase inhibitors (TKI) in *BRAF*^*WT*/*V*600*E*^-PTCs by secreting trombospondin (TSP-1) and TGF-β (157).

### Promising Target Therapy Approach for TME Cells in Thyroid Tumor

New treatment approaches have focused on the development of specific inhibitor compounds and neutralizing antibodies able to block chemokines, CAFs, TCSCs proliferation, and EMT. The aberrant activation of Sonic Hedgehog signaling is a common feature of ATCs, and it represents an important regulator of cancer cells and CAFs. Cancer cells secrete Shh ligand that encourages the peritumoral stroma, CAFs included, to produce a multitude of factors such as IGF, Wnt, and VEGF, which enhance cancer progression (158). In addition, this pathway is also activated in ATC and PTC cells in the absence of Shh in a Smo-dependent way through the downstream activation of Gli transcription factors caused by the oncogenic RAS/BRAF/MEK. Parascandolo et al. highlighted a specific positive correlation between stromal inhibition and reduction in TC progression. Coculture experiments and stimulation with conditioned medium showed that CAFs support TCP1 TC cells invasion, migration, and non-adherent growth abilities. These effects are both Smo and Shh dependent because they were abolished by the administration of cyclopamine and the 5E1 antibody. Furthermore, an increased Shh secretion has been observed in fibroblasts treated with conditioned medium derived from TC cells highlighting a bidirectional paracrine cross-talk, suggesting that targeting Shh pathway in ATC cells as well as in TME, especially in CAFs, could be exploited as potential target therapy (159). Visciano et al. elegantly studied that CXCL8 is a pivotal mediator of stemness and EMT in TC cells (77). Liotti et al. showed the mechanism through which CXCL8 exerts its autocrine function. The binding of CXCL8 to its receptor, CXCR1, is responsible for the formation of thyrospheres, self-renewal, and tumor-initiating ability. The identification of this autocrine protumorigenic loop could represent a therapeutic approach. Consistently, the treatment with the anti-CXCL8 blocking antibody decreases number and diameter of thyrospheroids, while anti-CXCL1 antibodies did not exert any effect on sphere formation (160). Furthermore, Liotti et al. studied the effect of reparixin, a CXCR1 receptor inhibitor. This treatment dramatically decreases cell survival, proliferation, EMT, and stemness of different TC cell lines (161).

In the last 10 years, different studies have been focused on the development of low molecular weight inhibitor drugs targeting EMT-initiating factors. The combination of BRAF inhibitors, dabrafenib, and MEK1/2 inhibitor, trametinib, have been suggested as a therapy for recurrent TCs (162). Wnt/β-catenin signaling pathway are involved in the EMT program of TC cells, and its inhibition could represent a potential therapy. Indeed, Hardin et al. found that the silencing of β-catenin reduces EMT markers expression in TC (21). Moreover, Gao and Han reported that the silencing of C-Met/PI3K/AKT pathway reverses EMT and metastasis of TC cells (163). The most promising treatments targeting TME in TCSCs are shown in Figure 4.

## Concluding Remarks

The gold standard therapy for all kinds of TCs is surgery associated with radioactive iodine (RAI) therapy. Unfortunately, a group of “advanced thyroid cancers” including <10% of differentiated TCs, many MTCs, as well as ATCs are not responsive to the standard therapeutic approach and evolve in distant metastatic sites. The 5-years survival rate is <50% in these patients compared with iodine-sensitive well-differentiated TC patients (110). Specific therapeutic targets have been identified for treatment of RAI refractory thyroid tumors. Several genetic alterations in the main molecular pathways, related with TC progression, such as *BRAF* and *RAS* mutations and *RET/PTC* gene rearrangements have been identified and allowed to develop therapeutic strategies for TC patients. To date, FDA has approved the use of different kinase inhibitor drugs, targeting the MAPK pathway (MKIs), such as Levatinib and Sorafenib for advanced RAI-R well-differentiated TCs and Cabozantinib and Vandetanib for MTCs. Due to their systemic toxicity and the ability by tumors to activate alternative proliferation pathways, these drugs partially induce beneficial effects. Moreover, the conventional therapy targets only the proliferating CSCs but no CSCs in a quiescent or slow proliferative state. TCSCs are responsible for TC initiation, progression, relapse, and metastatic dissemination. Given their plasticity, TCSCs are able to remain in a quiescent state within the TME niche. This can explain their persistence after the conventional therapies such as chemo- and radiotherapy. Therefore, a comprehensive knowledge of TCSCs biology may lead to the development of more effective therapeutic strategies. Efficient therapeutic approaches need to be explored to target both TCSCs and their progeny. Immunotherapy and epigenetic drugs in combination with standard therapy could represent promising strategies for TC treatment.

## Author Contributions

VV, FV, ML, and MT conceptualized and wrote the manuscript. CD'A, GP, AT, MG, SF, DG, and LM contributed to draft the manuscript. All authors contributed to the article and approved the submitted version.

## Conflict of Interest

The authors declare that the research was conducted in the absence of any commercial or financial relationships that could be construed as a potential conflict of interest.
